# A Case of a Congenital Cholesteatoma Without Growth for a Long Term

**DOI:** 10.7759/cureus.40945

**Published:** 2023-06-25

**Authors:** Yuichi Teranishi, Yuki Koda, Yasuyuki Kajimoto, Masaya Oishi, Kishiko Sunami

**Affiliations:** 1 Otolaryngology - Head and Neck Surgery, Osaka Metropolitan University, Osaka, JPN

**Keywords:** ossicular chain anomaly, spontaneous regression, closed-type, wait and scan, endoscopic ear surgery, congenital cholesteatoma

## Abstract

Congenital cholesteatoma is typically an expanding cystic mass of keratinizing squamous epithelium located medial to the intact tympanic membrane in patients with no prior history of perforation, otorrhea and ear. It is generally thought to be a progressive disease and is usually surgically removed upon detection as the first-choice treatment. As such, it is rare to be observed for a long term without progression. Here we report a rare case of congenital cholesteatoma that remained in an undetectable size and did not deteriorate mild hearing loss for 12 years. A seven years old boy was referred to us with right hearing impairment. Pure-tone audiometry found conductive hearing loss with an air-bone gap of 25 dB and a high-resolution computed tomography (CT) scan found the eroded long process of incus but did not detect any soft tissue density indicating congenital cholesteatoma. He initially did not wish to undergo surgery. His hearing level and image finding remained virtually unchanged during the next 12 years of the follow-up period. Twelve years later, endoscopic ear surgery was performed, which revealed a very small cholesteatoma mass, an eroded long process of the incus and ossicular chain discontinuities. We suspect that the cholesteatoma was originally larger, partially eroded the incus, then regressed to a very small size, and remained small for at least 12 years under our observation.

## Introduction

Congenital cholesteatoma is typically an expanding cystic mass of keratinizing squamous epithelium located medial to the intact tympanic membrane in patients with no prior history of perforation, otorrhea and ear surgery [1,2]. Congenital cholesteatoma accounts for 10-28% of pediatric cholesteatoma cases and 2-5% of all cholesteatoma cases, although this may be an underestimate [3]. Congenital cholesteatoma is generally thought to be a progressive disease where there is usually a greater aggressive growth activity in children than that in adults [4]. It may exist silently for a long time and then manifest with hearing loss (conductive or mixed), tinnitus, facial paralysis, intracranial inflammation, or external canal wall erosion [5]. On the other hand, there are reports of spontaneous regression in rare cases [6,7]. Most are a cystic form (closed-type congenital cholesteatoma (CTCC)) but there is also a membranous form that does not form a cyst (open-type congenital cholesteatoma (OTCC)) [8]. Pathologically, CTCC might be characterised by cystic structures surrounded by fibrous stroma and lined by squamous epithelium with a scarce number of inflammatory cells, whereas OTCC might be characterised by ruptured cyst or fragments composed of squamous epithelium and fibrous connective tissue with inflammation [9]. Here we report a rare case of congenital cholesteatoma that remained in an undetectable size by computed tomography (CT) scan and did not deteriorate mild hearing loss for 12 years.

## Case presentation

**Figure 1:**
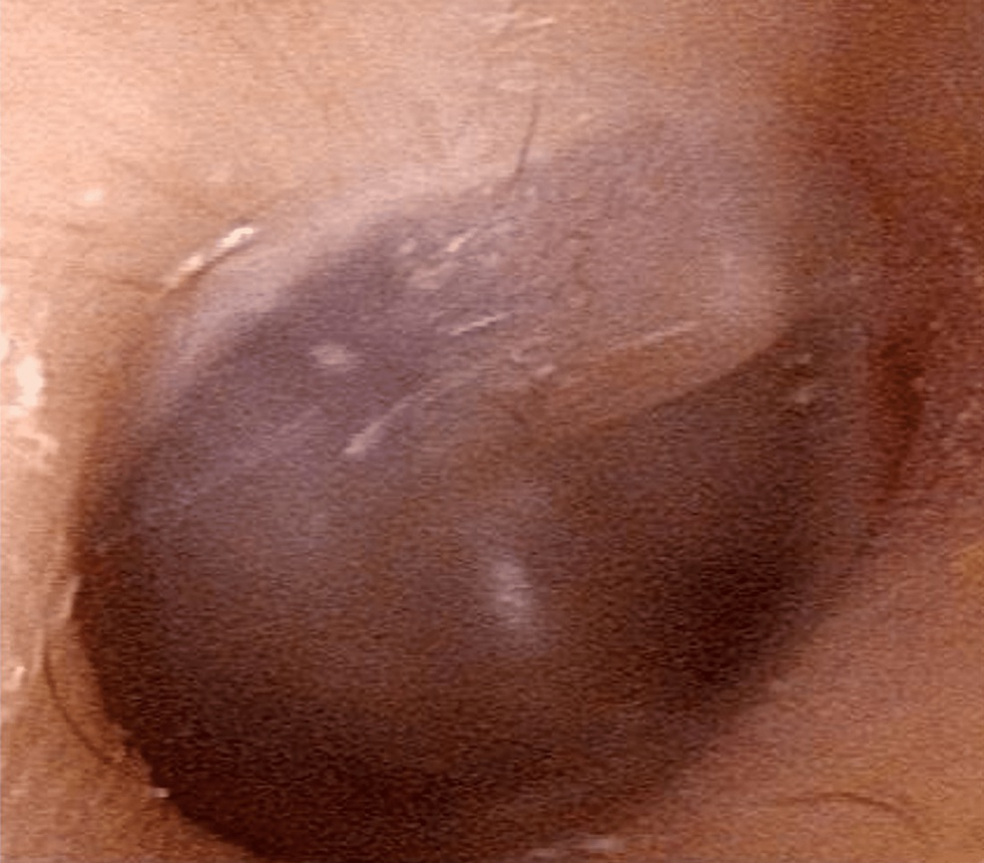
Otoscopic finding of the right ear of this case at age seven. The tympanic membrane and outer ear canal appear normal.

A seven years old boy was referred to our hospital for hearing loss in the right ear. His right ear hearing impairment was first detected during a health examination at school. The patient had no history of otitis media, otorrhea, ear trauma, otologic surgery and subjective hearing loss. Otomicroscopic examination revealed a normal tympanic membrane and outer ear canal (Figure 1).

**Figure 2:**
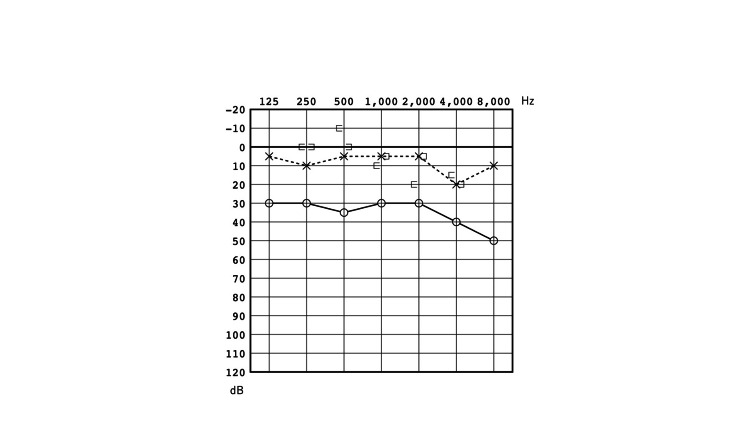
Pure tone audiogram at age seven. Pure tone audiogram at age seven showing conductive hearing loss with an air-bone gap of 25 dB on the right side and normal hearing of the left ear.

**Figure 3:**
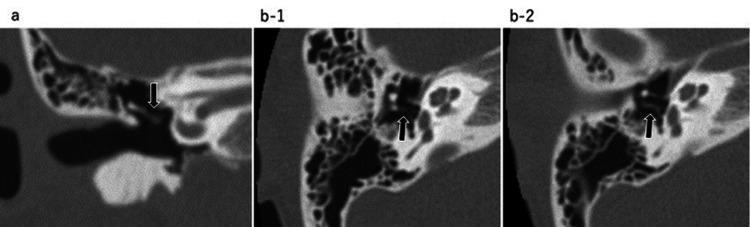
Temporal bone CT at age seven. a: Coronal view: The image of the long process of the incus is obscure (black arrow). b: Axial view: The image of the long process of the incus is obscure (black arrows). There is no sign of soft tissue densities indicating congenital cholesteatoma. CT: computed tomography

Pure-tone audiometry revealed conductive hearing loss with an air-bone gap of 25 dB (Figure 2). A high-resolution computed tomography (HRCT) scan of the temporal bone (120 kVp, high-resolution matrix (512 × 512), section thickness 0.5 mm, FOV 15-20 cm) showed that the long process of the incus was missing. There were no signs of soft tissue densities indicating congenital cholesteatoma (Figure 3). Although uncertain, an ossicular chain anomaly or a spontaneous regression of congenital cholesteatoma was a possible diagnosis. With fully informed consent and the patient's wishes not to operate at the time, we decided to take a “wait and scan” approach. During the follow-up period, pure-tone audiometry and CT scans were performed once every 2-3 years and no notable changes were detected for the next 12 years.

**Figure 4:**
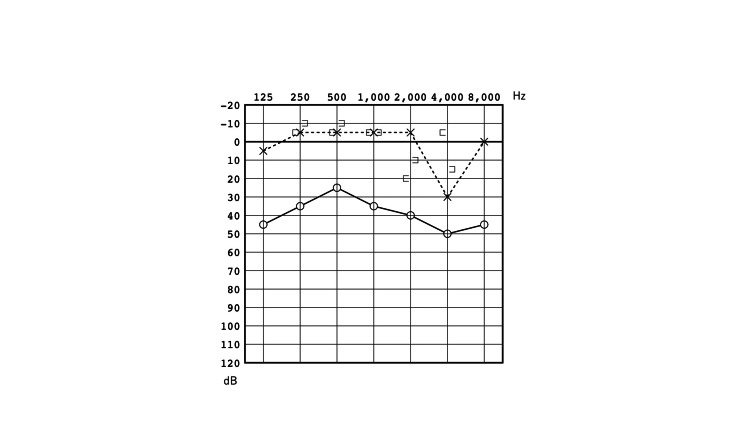
Pure tone audiogram at age 19. Pure tone audiogram of this case at age 19 showing conductive hearing loss with an air-bone gap of 31.6 dB on the right side and normal hearing of the left ear. Note no significant changes since 12 years ago (Figure 2).

**Figure 5:**
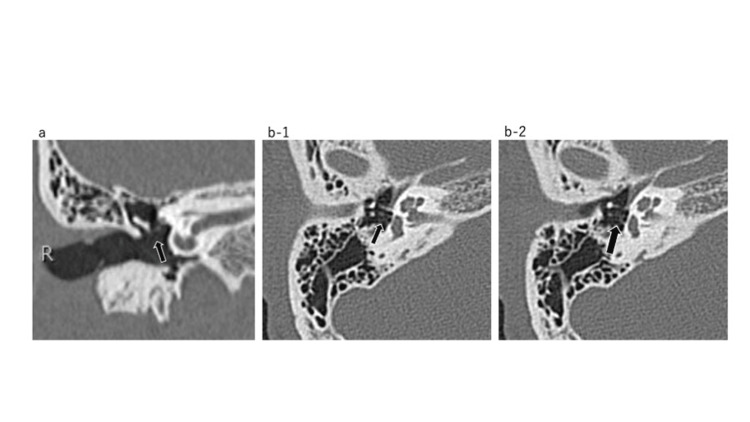
Temporal bone CT at age 19. a: Coronal view: The incudostapedial joint is discontinued (black arrow). b: Axial view: Note no significant changes since 12 years ago (Figure 3). CT: computed tomography

**Figure 6:**
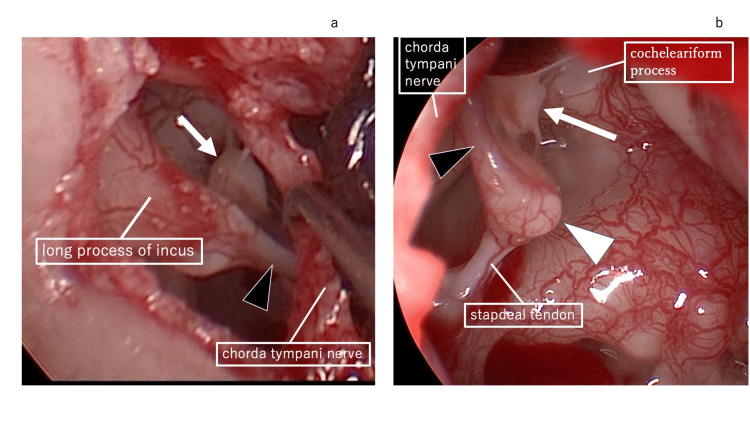
Surgical findings a: Surgical findings showing missing incudostapedial joint. The black arrowhead is the fibrous tissue connecting the long process of the incus and stapes. The white arrow is a small white mass of tissue attached to the anterior crus of the stapes. b: White arrow is the small white mass located between the stapes and the cochleariform process, and connected to the cochleariform process with thin fibrous tissue. Black arrowhead is the long process of the incus with a missing tip replaced by fibrous tissue. White arrowhead is the lenticular process that remained.

**Figure 7:**
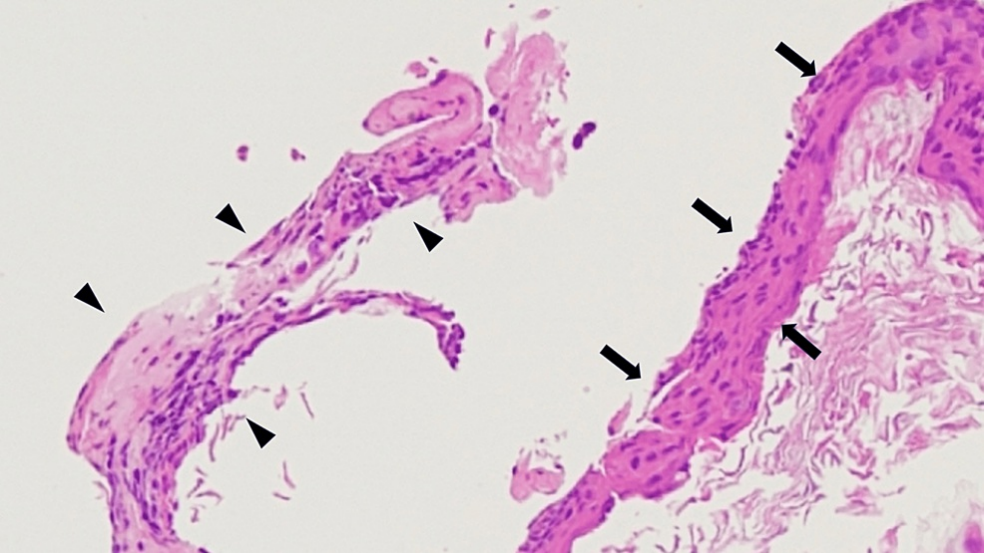
Pathological findings Cystic structure surrounded by fibrous stroma (arrow heads) and lined by squamous epithelium with a scarce number of inflammatory cells, characterizing the closed type. Note that the fibrous stroma and the squamous epithelium were damaged and detached during specimen preparation.

At age 19, after 12 year follow-up period, the patient wanted to improve his right ear hearing and underwent endoscopic surgery. A preoperative audiogram found that the conductive hearing loss in his right ear with an air-bone gap of 31.6 dB was not significantly different from 12 years ago (Figure 4). Preoperative HRCT (0.5mm slice) scans showed discontinued incudostapedial joint but no sign of soft tissues indicating cholesteatoma (Figure 5). Surgical findings included that the tip of the long process of the incus was eroded but the lenticular process was intact and attached to the head of the stapes while being connected to the incus body with fibrous tissue (Figure 6). Surprisingly, a small cystic form white mass was attached to the anterior crus of the stapes and connected to the cochleariform process with tiny fibrous tissue (Figure 6). The debris was covered by the matrix but was not present outside the matrix. Pathologic examination found keratin and a cystic structure surrounded by fibrous stroma and lined by squamous epithelium with few inflammatory cells (Figure 7), which led to the diagnosis of CTCC [8]. The CTCC was removed completely. The stapes were normal, so tympanoplasty type 3C was performed.

No recurrence has been acknowledged for more than three years.

## Discussion

We presented a rare case of CTCC which did not progress and did not deteriorate hearing for over 12 years. It is rare to observe such a long term without disease advancement, as congenital cholesteatoma is generally expected to grow and extend over time and is usually surgically removed upon detection as the first-choice treatment [10]. Although CTCC is usually visible as a whitish mass with otoscopy, the CTCC found, in this case, was so small and remained small for 12 years that it had been preoperatively undetectable with otomicroscopy and high-resolution CT of 0.5 mm slices.

We suspect that the cholesteatoma was originally larger, partially eroded the incus, then regressed to a very small size, and remained small for at least 12 years under our observation. The intact lenticular process and partially eroded long process of incus suggested that the ossicular chain discontinuity was caused by erosion associated with cholesteatoma rather than anomalies. How and when such regression could take place is uncertain. Possible hypotheses in literature we could relate to include spontaneous regression or disappearance of cholesteatoma [7] especially in the absence of inflammation as a result of the self-cleansing process [6] reported in OTCC [7].

Although the clinical and pathological findings were consistent with the diagnosis of CTCC, it is important to note other possibilities. We cannot rule out the possibility that the mass is a ruptured cyst and could be classified as OTCC. Slow growth may not be unusual in some OTCCs [11]. Another possibility is that this is a concurrent case of congenital cholesteatoma and congenital ossicular anomalies [12,13]. Pathophysiology of both types of CC and its association with congenital anomalies is still controversial today and an area of active research [8,13].

Endoscopic ear surgery provides superior visualization in a less invasive manner afforded by 0° and angled endoscopes [14]. It contributed to detecting small cholesteatoma in this case. As endoscopic ear surgery becomes more common, it is expected that our capability for differential or concurrent diagnoses for congenital cholesteatoma and ossicular anomaly will be also enhanced. Although the CC reported here did not advance for the long term, removal should be prioritized because of the possible acute exacerbation.

## Conclusions

In this case, we were able to observe the patient with CTCC before the operation for 12 years, during which time there was no worsening of the disease. In the absence of infection or other aggravating factors, congenital cholesteatoma may not worsen but removal should be prioritized because of the possible acute exacerbation.
